# The Expression of Aroma Components and Related Genes in Merlot and Marselan Scion–Rootstock Grape and Wine

**DOI:** 10.3390/foods11182777

**Published:** 2022-09-09

**Authors:** Chan Li, Hao Chen, Yiran Li, Tiantian Du, Jia Jia, Zhumei Xi

**Affiliations:** 1 College of Enology, Northwest A&F University, Yangling, Xianyang 712100, China; 2 Shaanxi Engineering Research Center for Viti-Viniculture, Yangling, Xianyang 712100, China

**Keywords:** scion–rootstock, volatile-related gene expressions, C6 compounds, LOX pathway

## Abstract

Rootstocks were bred and selected from several species in order to enhance the resistance against biotic or abiotic stresses. There are few studies on the effect of rootstocks on aroma and related gene expression. This study focused on the effects of three rootstocks, Kober 5BB (5BB), 1103 Paulsen (1103P), and Selection Oppenheim (SO4), on the aroma and volatile-related gene expression levels of Merlot and Marselan berries and wines. These three rootstocks reduced the total aroma content of Merlot wine. 5BB upregulated *VvLoXA* and showed increased C6 alcohols. 1103P enhanced the linalool from Merlot berry, with marked upregulation of *VvLinNer1*. Conversely, rootstocks increased the total aroma content of Marselan berry, verified by the related expression levels of volatile-related genes. For Marselan berry, 5BB and 1103P upregulated five *VvGTs* and nine genes from the LOX and MEP pathway. 1103P increased the contents of C6 alcohols, C6 aldehydes, and citronellol from Marselan berry. Compared to 5BB and SO4, rootstock 1103P provided berries of better quality and richer aroma volatiles to Merlot and Marselan, while all three of the rootstocks had a significant effect on scion–rootstocks.

## 1. Introduction

A scion–rootstock is a combination of the root system (rootstock) of one plant and the shoot (scion) of another [1]. The promotion of grape rootstocks began with phylloxera in Europe. Rootstocks were bred and selected from several species in order to enhance the resistance of the plant against biotic stresses, such as pathogens, and abiotic stresses, such as drought. Grape rootstocks are mostly derived from *V. riparia*, *V. berlandieri*, and *V. rupestris*. For example, rootstocks 5BB and SO4 are obtained from *V. riparia* and *V. berlandieri* and rootstock 1103P is obtained from *V. rupestris* and *V. berlandieri*. They are all primary rootstock cultivars in recent years [2,3]. Whereas SO4 shows excellent resistance to wet and acidic soil, 1103P is resistant to drought and saline, alkaline, and calcareous soil. 5BB exhibits high resistance to nematodes, strong growth, and suitability to dry deep soil [4]. On the one hand, rootstocks endow scion–rootstocks with multiple resistance. On the other hand, rootstocks have extensive effects on scion phenotype, nutrient absorption, yield, and berry quality [5,6,7,8,9]. The yield of Flame seedless increased after it was grafted onto rootstock Couderc 1613, Freedom, Harmony, Paulsen 1103, Richter 99, Richter 110, and Ruggeri 140 [6]. Merlot grafted onto rootstock SO4 accumulated higher levels of total proanthocyanidins in skins and seeds [5]. Cheng et al. found that rootstock Saltcreek and Macadams enhanced the anthocyanins concentration of Red Alexandria [7].

Aroma is a crucial substance that involves volatile alcohol, aldehyde, ketone, ester, and other organic components and helps estimate grape and wine quality [10]. According to the source, wine aroma is divided into three main groups: grape aroma, fermentation aroma, and maturation aroma [11]. The main components of grape-derived aroma are terpenes, norisoprenoids, methoxypyrazines, aliphatics, mercaptans, and phenylpropanoids, which are mostly formed via the mevalonic acid (MVA) pathway, the plastidial methylerythritol phosphate (MEP) pathway, and the lipoxygenase (LOX) pathway [12]. The MEP pathway, localized in plastids, synthesizes isopentenyl pyrophosphate (IPP) and dimethylallyl pyrophosphate (DMAPP) from pyruvate and glyceraldehyde 3-phosphate (G3P) via 1-deoxy-D-xylulose-5-phosphate synthase (DXS), 1-deoxy-D-xylulose 5-phosphate reductoisomerase (DXR), 1-hydroxy-2-methyl-2-(E)-butenyl-4-diphosphate reductase (HDR), etc. The MVA pathway, which occurs in the cytosol, brings acetyl-CoA into IPP and DMAPP [13]. These two isomeric five-carbon precursors generate GPP, FPP, GGPP, and β-carotene. Terpenes (monoterpenes, sesquiterpenes, and diterpenes) are synthesized by terpene synthase based on GPP, FPP, and GGPP, while β-carotene ulteriorly formed norisoprenoids precursor. Norisoprenoids, derived from the oxidative breakdown of carotenoids, which consist of GGPP, are the dominant volatile compounds of neutral cultivars, whereas terpenes contribute aroma mostly to muscat/floral cultivars [14]. The lipoxygenase pathway, the primary aroma source of non-muscat grapes, generates C6 and C9 volatile compounds from linoleic/linolenic acid via lipoxygenase (LOX), hydroperoxides (HPL), alcohol dehydrogenase (ADH), and alcohol acyltransferases (AAT).

The factor impacting grape volatiles is diversity, which depends on cultivar, soil, weather, water, training and canopy management, fertilization treatment, etc. [15]. Different cultivars have different characteristic grape aromas, such as 1,1,6-trimethyl-1,2-dihydronaphthalene (TDN) for Riesling and terpenes for Muscat cultivars. Ji et al. [16] found Traminette to have different aroma compounds between the cool site and the hot site and the cool site to have higher 6-carbon aldehydes and the hot site to have higher monoterpenes. Wang et al. (2019) suggested that rootstock SO4 is not recommended because the cabernet sauvignon berries grafted onto SO4 have a lower concentration of total esters compared to berries from own-rooted samples, while 101-14, Ganzin 1, 110R, and 5BB increase the concentrations of C13-norisoprenoids [17]. Carrasco et al. (2020) reported that rootstocks 99R, 140Ru, 110R, 1103P, and Gravesac lead to a higher content of total ethyl esters in Merlot wines, while the own-rooted wines have the lowest content of *(E)*-3-hexenol and total ethyl esters [18]. In vineyards, grafting is a common cultivation method. Nonetheless, there are few studies on the effect of rootstocks on aroma and related gene expression. In this study, we used two scions (Merlot and Marselan, *Vitis vinifera* L.) and three rootstocks (5BB, 1103P, and SO4) and investigated the aroma characteristics of grapes and wines from different scion–rootstocks, as well as the diversity of volatile compounds and their correlation with related gene expression. The objective of this work was to identify the impact of rootstocks on the aroma profiles and related gene expression of different scion–rootstock grapes and wines and provide further insight into the quality changes due to cultivation.

## 2. Materials and Methods

### 2.1. Location and Material

The specimens were collected during the 2021 growing season from a commercial vineyard located in Chateau Yaoking, Xiangfen County, Shanxi Province, China (111°34′25.77″ E, 35°55′42.21″ N). The experimental materials, including Merlot and Marselan grapevines (*Vitis vinifera* L.), both own-rooted ones and those grafted onto Kober 5BB (5BB), 1103 Paulsen (1103P), and Selection Oppenheim (SO4) rootstocks (MT, MT-5BB, MT-1103P, MT-SO4, MN, MN-5BB, and MN-1103P), were planted at 1.5 m × 3.0 m (in-row × between-row spacing, respectively) in 2013 (8 year-old). Before the materials were planted, the planting ditch (80 cm) was filled with a mixture of surface mellow soil, straw, and organic fertilizer (150 m^3^/ha) for soil replacement. The winery is located in a temperate continental climate, with a long frost-free period and little rainfall. The grapes required no additional irrigation.

The experimental design was a randomized complete block design, with three blocks and two vines (replications) per block. All samples were harvested on 5 September 2021 (*EL*-38). The seven treatments were replicated three times in randomized blocks, with three rows per replication.

### 2.2. Winemaking

At harvest, 20 kg of sound grapes from each replicate were destemmed and crushed to obtained the must, which was treated by adding 60 mg/L of SO_2_. The must was placed in 10 L tanks, 30 mg/L of pectinases (VINOCLEAR CLASSIC, LAMOTHE-ABIBT, French) was added, and then the mixture was allowed to undergo pre-fermentative maceration for 36 h. Then, the enological parameters of the grape must was analyzed and it was inoculated with 200 mg/L of activated commercial yeast strain (FRF, Enartis, Italy). The fermentation temperature was controlled at 21 ± 1 °C. Must density (hydrometer) and temperature were measured three times a day until the reducing sugar was lower than 4 g/L. The reducing sugar and the density of the must were determined according to the National Standard of People’s Republic of China (GB/T15038-2006). Then, the must was treated with 100 mg/L SO_2_ to stop the alcoholic fermentation, grape skins were pressed, and the lees removed manually to obtain the wine. After that, the wine was racked in sealed glass containers (5 L), protected from light, and stored for further clarifying and aging for 3 months (5 ± 1 °C).

### 2.3. Enological Parameter Analysis

Physicochemical parameters, including total soluble solids, pH, titratable acidity (g tartaric acid/L), and total sugar of grapes, and the alcohol degree of the wines were measured according to OIV methodologies (OIV-MA-INT-00-2021: https://www.oiv.int/en/technical-standards-and-documents/methods-of-analysis/compendium-of-international-methods-of-analysis-of-wines-and-musts, accessed on 10 August 2022). Lactic acid, tartaric acid, glycerol, glucose, and fructose were analyzed by Lyza 5000 Wine (Anton Paar, Graz, Austria). For each replicate of the seven treatments, each chemical analysis was replicated in triplicate.

### 2.4. Volatile Compounds SPME and GC-MS Analysis

Grape and wine volatile compounds were analyzed with some modifications as described by Wang et al. [19]. Grape berry juice was extracted as described Yue et al. [20]. Powder samples from the frozen grape berries without seeds and stems were stored at 4 °C for 6 h to clarify with centrifugation (10,000 rpm, 10 min). Briefly, a 15 mL glass phial containing 8 mL of the sample (6 mL of wine to which 2 mL of pure water was added; 8 mL of pellucid grape juice) and 2 g of sodium chloride mixed with internal standard (40 mg/L, 2-octanol) and a magnetic stirring bar was dipped in a thermostatic water bath to equilibrate for 15 min at 40 °C. The volatile contents were measured via headspace phase microextraction combined with gas chromatography and mass spectrometry as described by Wang et al. [21]. The solid-phase micro-extraction (SPME) fiber coating was divinylbenzene/carboxen/polydimethylsiloxane (DVB/CAR/PDMS, 50/30 μm, 2 cm StableFlex/SS) procured from Bellefonte (PA, USA).

SPME was performed with a magnetic stirrer (40 °C, 30 min), followed by the desorption of the analytes into the gas chromatograph injector (3 min). The GC–MS analysis system was a TRACE DSQ single quadrupole (Thermo-Finnigan, San Jose, CA, USA); the analytical column was a DB-Wax capillary column (30 m × 0.32 mm i.d., 0.25 μm film thickness), J&W (Folsom, CA, USA); the carrier was He (flow rate of 1 mL/min); the transfer line temperature was 230 °C; the injection temperature was 250 °C; the ion source temperature was 230 °C; and the temperature program was as follows. The system was initially at 40 °C for 3 min. The temperature was then increased to 160 °C at 4 °C/min and then to 230 °C at 7 °C/min and maintained for 8 min. Mass spectra were recorded in electron impact (EI) ionization mode. Mass spectrometry: mass range, 33–450 amu, scanned at 1 s intervals [21].

### 2.5. Gene Expression Analysis by qRT-PCR

Total RNA was extracted from frozen grape samples using an RNA extraction kit (Bioteke, Beijing, China). The total RNA concentration was determined, and the RNA quality was assessed using a spectrophotometer (Biodrop, Cambridge, UK) and by agarose gel electrophoresis. The purified RNA (1 μg) was then used as the template to synthesize cDNA with the cDNA synthesis kit (Vazyme Biotech, Nanjing, China). The expression levels of specific genes were determined by quantitative real-time PCR (qRT-PCR) with the QuantStudio 6 (Life Technologies, Carlsbad, CA, USA). Primers for *VvDXS1*, *VvDXS3*, *VvDXR*, *VvHDR*, *VvGPPS*, and *VvFPPS* were from a previous study by [13], whereas those for *VvGT7*, *VvGT14*, *VvGT9*, *VvGT5*, and *VvGT6* were from [22,23]. Finally, the primers specific for *VvActin*, *VvCCD4a*, *VvCCD4b*, *VvCCD1*, *VvLinNer1*, *VvLinNer2*, *VvLOXA*, *VvADH1*, *VvADH2*, and *VvADH3* were designed using NCBI. The *VvActin* gene was used as a reference control. Gene expression levels were determined with the 2^−ΔΔCt^ method, with the first control own-rooted Merlot and Marselan sample serving as the reference.

### 2.6. Data Analysis

The significance of differences between treatments for grape/wine enological parameters and volatile composition was analyzed by a one-way ANOVA followed by the Tukey test (*p* ≤ 0.05) with SPSS 24.0 (SPSS Inc., Chicago, IL, USA). The data are provided in the tables and figures herein as the mean ± standard deviation.

## 3. Results

### 3.1. Effects of Rootstocks on Berry and Wine Physicochemical Parameters

The physicochemical parameters of Merlot and Marselan berries and wines from different rootstocks are presented in Table 1 and Table 2. Berries from the Merlot-1103P scion–rootstock (MT-1103P) showed higher levels of total sugar (TS) and total soluble solids (TSS) than those from Merlot-5BB (MT-5BB) and Merlot-SO4 (MT- SO4) scion–rootstocks and the merlot own-rooted scion (MT), which directly affected the alcohol degree of the wine. Observably, the fructose content of berries from MT-5BB, MT-1103P, and MT-SO4 was higher than that from MT, while that of berries from MT-1103P was the highest. For all the Merlot scions, the titratable acidity of berries from the rootstock was significantly higher than that of berries from the own-rooted samples. With respect to pH and glycerol, wines from the three Merlot scion–rootstocks showed higher levels than wines from own-rooted samples, with rootstock 1103P showing the most significant level. At the same time, 1103P lowered the tartaric acid and titratable acid content of Merlot wine compared with other rootstocks and own-rooted samples. The phenomenon had also been reported in Monastrell [24]. Compared to the Marselan own-rooted scion (MN), rootstocks 5BB and 1103P (MN-5BB, MN-1103P) endowed berries with lower contents of TS, TSS, fructose, and glucose. With regard to titratable acidity in berries, MN-5BB and MN-1103P showed higher levels than MN, while MN-5BB showed the highest level. MN-5BB wine presented a higher level of tartaric acid than MN and MN-1103P. However, wines from Marselan scion–rootstocks presented lower pH and glycerol than wines from own-rooted samples.

### 3.2. Effects of Rootstocks on Merlot Berry and Wine Volatile Composition

Table 3 lists the concentrations of the main aroma compounds in the Merlot scion–rootstocks grapes and wines. C6 volatile compounds have been reported as characteristic compounds for non-muscat grape cultivars mainly composed of two aldehydes, five alcohols, three esters, and one acid [25]. As the most abundant volatile compounds in grapes, hexanal and *(E)*-2-hexenal are two dominant C6 aldehydes from linoleic/linolenic acid in the LOX pathway. They were found to be greenish, with a fruity flavor, and to significantly influence the aromatic feature of the grapes [26,27]. Regarding the C6 aldehydes, hexanal and *(E)*-2-hexenal were found in MT and MT-5BB grapes but appeared in all Merlot wines. Wine from own-rooted Merlot was shown to have the highest hexanal and *(E)*-2-hexenal levels. With three Merlot scion–rootstocks, MT-1103P presented a higher level than MT-5BB and MT-SO4. Concerning C6 alcohols, MT-1103P and MT-SO4 grapes had only hexanol, while MT and MT-5BB grapes had hexanol, *(Z)/(E)*-3-hexenol, and *(E)*-2-hexenol. These three C6 alcohol profiles were all found in wines from MT, MT-5BB, MT-1103P, and MT-SO4. Additionally, these Merlot scion–rootstock wines contained higher contents of *(E)*-2-hexenol than *(Z)/(E)*-3-hexenol. Similar observations were made in other grape cultivars [27,28,29]. Meanwhile, MT-5BB and MT-SO4 had a higher *(E)*-2-hexenol level than MT whereas MT-1103P showed the lowest level. This differed from the results of a study that showed wine from own-rooted Merlot with the lowest content of *(E)*-3-hexenol and the highest content of hexanol and *(Z)*-3-hexenol [18]. Rootstocks gave the Merlot grapes a markedly higher hexanol than own-rooted Merlot, which was also manifested in wines. C6 esters, as important volatiles, have been depicted as fruity, floral, and sweet flavors in fruits [30,31]. Isobutyl acetate, the only C6 ester, was found in MT-1103P and MT-SO4 scion–rootstocks grapes. Hexanoic acid was found in Merlot scion–rootstock grapes and wines, the same as in a past study [27]. Wines from MT-5BB and MT-SO4 had higher hexanoic acid levels than MT and MT-1103P when the C6 acid was present only in MT and MT-5BB grapes. At the same time, MT-1103P and MT-SO4 grapes contained another C6 acid, named butanoic acid, and ethyl ester.

Monoterpenes, which directly affect the floral aroma of Muscat varieties, were less in non-muscat grapes such as Merlot. Rootstock 1103P and SO4 significantly induced the synthesis of citronellol in the Merlot grape, with the exception of linalool and geraniol. Among the monoterpenes for wines, MT and MT-1103P had higher geraniol than MT-5BB, while MT-SO4 had the highest linalool.

Among these compounds, 5BB had a negative influence on ester concentrations of Merlot grape, while 1103P increased the concentrations of alcohol, ketone, ester, and acid significantly more than own-rooted Merlot. Meanwhile, SO4 statistically enhanced alcohol and acid concentrations in Merlot grape. The most abundant aroma profile in Merlot berries was significantly different: in MT and MT-5BB, aldehydes were responsible for the aroma, and in MT-1103P and MT-SO4, alcohol was responsible for the aroma. However, the alcohol concentrations of Merlot wines grafted onto 5BB, 1103P, and SO4 had a flatter level compared with the alcohol concentration of own-rooted Merlot, while aldehyde concentrations were observably lower than those in own-rooted Merlot. Additionally, 5BB and SO4 showed a consistent positive influence on the accumulation of wine acids.

### 3.3. Effects of Rootstocks on Marselan Berry and Wine Volatile Composition

Table 4 lists the concentrations of the main aroma compounds of the Marselan scion–rootstock berries and wines. In Marselan berries, pentanoic acid was detected in large quantities and hexanol was also found at a high level. Notably, the major C6 compounds, such as hexanal, *(E)*-2-hexenal, and 3-hexenol, were not detected. Compared to the own-rooted berries, 5BB had significant positive effects on the concentrations of isobutyl acetate and 3-methyl-4-oxo-pentanoic acid. In contrast, C6 compounds were detected in Marselan wines. The rootstocks 5BB and SO4 changed the relative levels of the aroma volatiles of the eight types of Marselan wines. They had an improved influence with hexanal and *(E)*-2-hexenal compared to own-rooted wine when the concentrations of hexanoic acid and *(E)*-3-hexenol had decreased markedly. Additionally, a small amount of 2-ethyl-furan was found in wines from grafted Marselan, which offered a strong sweet and coffee-like aroma at low concentrations. The wine from MN-1103P had the highest *(E)/(Z)*-2-hexenol, while the wine from MN-5BB was found to have the lowest level of hexanol.

Compared to the Marselan grape, four terpenes were found in MN-5BB and MN-1103P, while only two terpenes were found in own-rooted berries. Citronellol and α-terpineol were found in all Marselan berries, and MN-1103P had a significant effect on both compounds. Citronellol afforded an elegant rose aroma, while α-terpineol led to a clove aroma. Farnesene and nerolidol were detected only in MN-5BB and MN-1103P grapes. MN-5BB had a substantial farnesene content, which endowed a green color and a fragrant aroma, while MN-1103P had a higher nerolidol, affording apple and rose aromas, along with a hint of wood aroma. A total of seven terpenes were found in Marselan wines: linalool, α-terpineol, geraniol, citronellol, trans-β-ocimene, neral, and β-myrcene. Citronellol was not detected in MN. Geraniol, linalool, and β-myrcene were the main volatile terpenes in the Marselan wines. While geraniol showed a warm, sweet rose aroma, linalool had a lily-of-the-valley aroma and β-myrcene presented orange and balsam aroma. MN-5BB had significant effects on the geraniol amount compared to MN and MN-1103P.

The concentrations of total esters in MN-5BB grapes were statistically higher than those in MN and MN-1103P, while the concentrations of total ketones in MN-1103P were significantly higher than in others. The abundant aroma profiles in Marselan berries and wines were different, with alcohol and acid being responsible for the aroma in berries and aldehyde and alcohol being responsible for the aroma in wines. Among these compounds, rootstock 5BB and 1103P had a negative influence on the concentrations of total alcohols and aldehydes, while MN-1103P berries had the lowest total acids. On the contrary, the total aldehydes in MN-5BB and MN-1103P wines had a dramatically higher level than in MN, while the total alcohols and ketones had a similar level in all Marselan wines. Specially, rootstocks had a negative influence on the acid concentrations.

### 3.4. Rootstocks Effects on Volatile-Related Gene Expression

**Figure 1:**
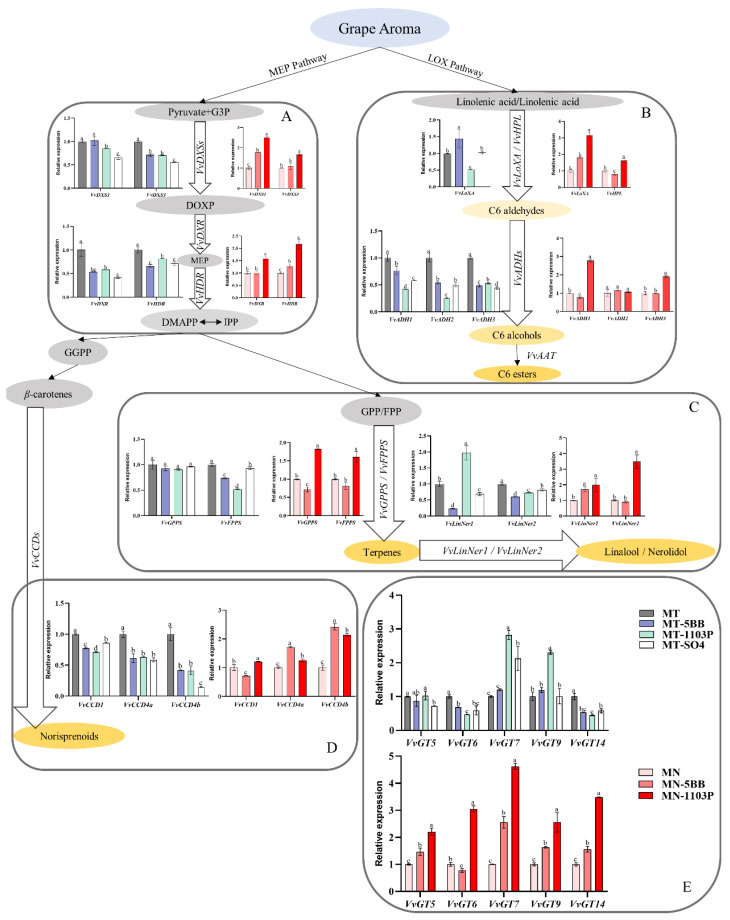
Change in the expression of the volatile-relative genes of Merlot and Marselan. (**A**) *VvDXS1*, *VvDXS3*, *VvDXR*, *VvHDR* from the MEP pathway. (**B**) *VvLoXA, VvADH1, VvADH2, VvADH3* from the LOX pathway. (**C**) *VvGPPS, VvFPPS, VvLinNer1, VvLinNer2* from the MEP pathway. (**D**) *VvCCD1, VvCCD4a, VvCCD4b* from the C13-compound synthesis pathway. (**E**) *VvGT5, VvGT6, VvGT7, VvGT9, VvGT14* for the synthesis of glycosylated volatile compounds. Data are the mean concentration (n = 3). Different letters show significant differences between treatments by Duncan’s multiple range test (*p* ≤ 0.05).

As a result of the observed rootstocks impacted in terms of volatile contents in grapes and wines, we analyzed the expression of the genes in aroma synthesis pathways by qRT-PCR. We selected five genes in the LOX pathway encoding the following enzymes: lipoxygenase (*VvLoXA*), alcohol dehydrogenase 1 (*VvADH1*), alcohol dehydrogenase 2 (*VvADH2*), and alcohol dehydrogenase 3 (*VvADH3*) (Figure 1B). The *VvLoXA*, *VvADH1*, *VvADH2*, and *VvADH3* expression levels of MT-1103P were significantly down-regulated, whereas these were markedly upregulated in MN-1103P. Meanwhile, rootstock 5BB significantly increased the expression of *VvLoXA* in both Merlot and Marselan grapes, while *VvADH1* and *VvADH2* were significantly down-regulated. Rootstock SO4 limited the expression of *VvADH1*, *VvADH2*, and *VvADH3* in Merlot grapes.

We selected eight genes in the MEP pathway encoding the following enzymes: 1-deoxy-D-xylulose-5-phosphate synthase (*VvDXS1*), 1-deoxy-D-xylulose-5-phosphate synthase (*VvDXS3*), 1-deoxy-D-xylulose 5-phosphate reductoisomerase (*VvDXR*), 4-hydroxy-3-methylbut-2-enyl diphosphate reductase (*VvHDR)* (Figure 1A), solanesyl diphosphate synthase 3 (*VvGPPS*), farnesyl diphosphate synthase (*VvFPPS*), (*3S*)-linalool/(*E*)-nerolidol synthase (*VvLinNer1*), and (*3S*)-linalool/(*E*)-nerolidol synthase (*VvLinNer2*) (Figure 1C). Compared with the levels in own-rooted samples, *VvDXS1*, *VvDXS3*, *VvDXR*, *VvHDR*, *VvFPPS*, and *VvLinNer2* expression levels in MT-1103P were decreased observably. Contrarily, the gene expression of the MEP pathway from Marselan was significantly upregulated. 5BB had similar effects to rootstock 1103P in Merlot in that *VvDXS3*, *VvDXR*, *VvHDR*, *VvFPPS*, *VvLinNer1*, and *VvLinNer2* were down-regulated, while *VvDXS1* and *VvGPPS* showed no obvious changes. Rootstock SO4 limited the expression of all genes of the MEP pathway except for *VvGPPS* in Merlot grapes. However, in Marselan scion–rootstocks, the *VvDXS1*, *VvHDR*, and *VvLinNer1* expression levels were upregulated, while *VvDXS3*, *VvDXR*, *VvFPPS*, and *VvLinNer2* had no difference compared to the levels in own-rooted samples. Especially, the gene expression of *VvGPPS* was decreased in MN-5BB.

We selected three genes in the C13-compound synthesis pathway encoding the following enzymes: carotenoid cleavage dioxygenase 1 (*VvCCD1*), carotenoid cleavage dioxygenase 4a (*VvCCD4a*), and carotenoid cleavage dioxygenase 4b (*VvCCD4b*) (Figure 1D). Rootstocks 5BB, 1103P, and SO4 significantly decreased the expression levels of *VvCCD1, VvCCD4a*, and *VvCCD4b* in Merlot. 1103P upregulated the expression levels of *VvCCDs* in Marselan, whereas 5BB upregulated the expression levels of *VvCCD4a* and *VvCCD4b* down-regulated that of *VvCCD1*.

We selected five genes of glycosylated volatile compounds encoding the following enzymes: anthocyanidin 3-*O*-glucosyltransferase (*VvGT5*), UDP-sugar flavonoid glycosyltransferase (*VvGT6*), UDP-glycosyltransferase (*VvGT7*), beta-D-glucosyltransferase (*VvGT9*), and 7-deoxyloganetin glucosyltransferase (*VvGT14*) (Figure 1E). Different rootstocks had different effects on Merlot GT gene expression: rootstock 5BB down-regulated *VvGT6* and *VvGT14*; rootstock 1103P upregulated *VvGT7* and *VvGT9* and down-regulated *VvGT6* and *VvGT 14*; and rootstock SO4 down-regulated *VvGT5, VvGT6*, and *VvGT14* and upregulated *VvGT7*. Except for *VvGT6*, the gene expression levels of all other *VvGTs*, including *VvGT5*, *VvGT7*, *VvGT9*, and *VvGT14*, were upregulated in Marselan-grafted 5BB, while 1103P upregulated the expression levels of all *VvGTs* in Marselan.

On the whole, rootstocks down-regulated the expression of aroma-related genes in Merlot grapes and SO4 was the most significant, followed by 5BB and 1103P. On the contrary, rootstocks upregulated the expression of aroma-related genes in Marselan grapes, and 1103P was better than 5BB.

## 4. Discussion

The influence of rootstocks on the TSS was measured, showing that the berries on grafted vines accumulated higher TSS than those on the own-rooted Merlot berries, which is inconsistent with previous reports [4,8]. However, TSS of Marselan berries was decreased by rootstocks (5BB and 1103P). In addition, the values of berry TSS/TA in scion–rootstocks MT-5BB, MT-1103P, MT-SO4, MN-5BB, and MN-1103P were lower than those in the own-rooted samples. This result is consistent with previous studies [8,32], which suggests that rootstock 101-14 could delay the maturity of Chardonnay berries. However, it is worth noting that this statement cannot be used as a single criterion to judge berry ripeness. Under the same condition, the TSS/TA levels of Merlot and Marselan scion–rootstocks were lower than those of the own-rooted samples. Rootstocks accelerated the ripening of Merlot berries when they significantly increased TSS and TA. Conversely, rootstocks hindered the ripening of the Marselan berries when they decreased TSS but increased TA levels. Rootstock had different effects on different scions.

Significantly, rootstocks (5BB, 1103P, and SO4) harmed the aroma of Merlot wine. Rootstocks markedly reduced the content of C6 aldehydes (hexanal and *(E)*-2-hexenal), increased the content of C6 alcohols (hexanol), and decreased the total amount of C6 compounds in Merlot wines. In addition, C6 volatile compounds were converted from acetate compounds to aldehydes and finally to alcohols during early, middle, and late berry developmental stages, respectively. In the later stages of berry development, alcohols dominated, followed by aldehydes [33]. The main aroma component of MT wine was aldehydes, which was different from Merlot scion–rootstocks, where the main aroma component was alcohol. Previous studies have determined that berry ripening stages by the alcohol–aldehyde ratio: alcohols usually have a higher herbaceous odor than related aldehydes [33,34]. The alcohol–aldehyde ratio of Merlot wines from scion–rootstocks was significantly increased, indicating that rootstocks promote berry ripening in Merlot, which may be one of the reasons why the total amount of aroma in grafted rootstocks was lower than that in own-rooted Merlot. Rootstocks had a certain effect on the terpene content, for example, the total amount of terpenes in MT-1103P was higher than that in MT, while the content of linalool in MT-SO4 was significantly higher than that in MT.

Meaningfully, rootstocks had a positive effect on the aroma of Marselan wine, indicating a significant increase in the total amount of C6 compounds. Rootstocks observably increased the content of C6 aldehydes, while rootstock 5BB decreased the content of C6 alcohols and rootstock 1103P increased the content of C6 alcohols. Rootstock 5BB markedly increased geraniol content, and the total terpene content of MN-5BB was higher than that of MN. Rootstocks significantly increased the aldehydes content of Marselan wine but decreased the alcohol–aldehyde ratio, which may be one of the characteristics of rootstock prolonging the ripening of Marselan. That might be one of the reasons why the total aroma of wines was higher in the grafted combination than in own-rooted samples.

In terms of total volatile compounds, rootstocks prominently reduced the aromas of Merlot and significantly increased the aromas of Marselan, consistent with the results of volatile-related gene expression levels. For Merlot, the expression of aroma genes in scion–rootstocks was generally lower than that in own-rooted samples, while the expression levels of *VvLoXA*; *VvGT7* from MT-5BB; and *VvLinNer1*, *VvGT7*, and *VvGT9* from MT-1103P were higher than those from own-rooted samples. This resulted in a generally negative impact of the rootstock on the volatiles in Merlot wines, which is consistent with previous studies [18]. At the same time, there were differences between the Merlot grape aroma substances and volatile-related gene expression, which is possibly related to the accumulation and state of volatile compounds in the early stage. Volatile aroma compounds in grapes are typically found both as free-form and bound-form. Previous studies have monitored volatile compounds and gene expression levels during the grape berry ripening stage and found that the period of high accumulation of aroma biosynthetic pathways is preveraison [26,27,33]. For Marselan, the expression of the volatile-related genes in the rootstocks was generally elevated, which resulted in a positive effect of wine grafted rootstocks, increasing the aroma concentration and berry quality. In general, rootstock 1103P had a better effect on Merlot and Marselan than other rootstocks. For Merlot and Marselan, we observed differences in C6 compounds and terpenes between berry and wine: volatile compounds were at higher levels in wine than in berries, which was related to the glycosylation of aromatic compounds. A previous study found that functionally characterized monoterpenol glucosyltransferases (GTs) had an effect on the in vitro catalytic activity in C6 alcohols [35].

## 5. Conclusions

Rootstocks had diverse effects on different scions, verified by the aroma and related-gene expression of Merlot and Marselan scion–rootstocks. Rootstocks 5BB, 1103P, and SO4 enhanced the fruit ripening of Merlot, increased the sugar and acid content, decreased the total amount of aroma components, and down-regulated the expression of aroma-related genes. Rootstock 1103P showed better performance compared with rootstocks 5BB and SO4. Rootstocks 5BB and 1103P restrained the ripening of Marselan grapes by reducing the sugar content and increasing the acid content. With the increase in the total amount of aroma components and the upregulation of aroma-related genes, rootstock 1103P performed better than 5BB. This finding may provide some basis for the selection of wine grape scion–rootstocks in different regions. For areas prone to rain at maturity, Merlot-1103P can be selected to shorten the grape maturity stage, ensuring there is little damage to aroma and good resistance to losses due to climate. The combination of Marselan-1103P, which has a positive effect on aroma abundance and content, can be selected to further improve the aroma and quality of wine in areas with little rainfall at maturity.

## Figures and Tables

**Table 1:** Effects of physicochemical parameters in Merlot and Marselan berries induced by the rootstocks (g/L).

	Grapes						
	MT	MT-5BB	MT-1103P	MT-SO4	MN	MN-5BB	MN-1103P
Total sugar	196.77 ± 0.42 b	191.87 ± 0.26 c	206.40 ± 0.64 a	187.47 ± 0.62 d	204.27 ± 0.53 A	197.23 ± 0.45 B	193.10 ± 0.43 C
Glucose	96.40 ± 0.78 a	86.70 ± 0.90 c	93.17 ± 0.54 b	84.60 ± 0.51 d	93.06 ± 0.48 A	90.03 ± 0.52 B	87.43 ± 0.52 C
Fructose	98.57 ± 1.09 c	103.17 ± 0.90 b	111.50 ± 0.78 a	101.13 ± 0.86 b	108.83 ± 0.59 A	105.40 ± 0.50 B	102.83 ± 0.52 C
Total soluble solids	219.07 ± 0.21 d	229.37 ± 0.53 b	248.40 ± 0.70 a	225.20 ± 0.99 c	244.37 ± 0.54 A	236.50 ± 0.64 B	231.13 ± 0.33 C
Titratable acidity	5.61 ± 0.06 b	6.53 ± 0.08 a	6.56 ± 0.03 a	6.58 ± 0.02 a	6.94 ± 0.04 C	7.85 ± 0.05 A	7.34 ± 0.10 B
TSS/TA	39.04	35.13	37.86	34.22	35.21	30.13	31.49

All parameters are listed with their standard deviations (n = 3). For each parameter, values with different letters are significantly different between the samples (*p* ≤ 0.05).

**Table 2:** Effects of physicochemical parameters in the Merlot and Marselan wines induced by the rootstocks.

	Wine						
	MT	MT-5BB	MT-1103P	MT-SO4	MN	MN-5BB	MN-1103P
Alcohol degree (%vol)	12.06 ± 0.05 B	11.78 ± 0.05 C	12.56 ± 0.08 A	12.23 ± 0.12 B	12.32 ± 0.05 a	9.65 ± 0.09 b	9.33 ± 0.08 c
Titratable acidity (g/L)	5.26 ± 0.04 B	5.43 ± 0.08 A	5.07 ± 0.05 C	4.95 ± 0.04 C	5.88 ± 0.04 a	5.46 ± 0.06 b	4.88 ± 0.05 c
Lactic acid (g/L)	1.61 ± 0.04 A	1.38 ± 0.04 B	1.64 ± 0.04 A	1.65 ± 0.03 A	2.11 ± 0.04 a	1.44 ± 0.02 b	1.92 ± 0.03 c
Tartaric acid (g/L)	1.29 ± 0.05 B	1.50 ± 0.00 A	1.14 ± 0.02 C	1.29 ± 0.02 B	1.34 ± 0.02 c	2.13 ± 0.02 a	1.77 ± 0.03 b
pH	3.62 ± 0.02 B	3.60 ± 0.01 B	3.73 ± 0.01 A	3.70 ± 0.01 A	3.73 ± 0.00 a	3.50 ± 0.01 c	3.64 ± 0.00 b
Glycerol (g/L)	8.00 ± 0.08 C	8.23 ± 0.09B C	8.77 ± 0.12 A	8.37 ± 0.12 B	7.90 ± 0.24 a	6.90 ± 0.14 b	6.83 ± 0.12 b

All the parameters are listed with their standard deviations (n = 3). For each parameter, values with different letters are significantly different between the samples (*p* ≤ 0.05).

**Table 3:** Volatile compounds of grapes and wines from own-rooted Merlot and Merlot grafted onto different rootstocks: Kober 5BB (MT-5BB), 1103 Paulsen (MT-1103P), and Selection Oppenheim (MT-SO4).

	Grape				Wine			
	MT	MT-5BB	MT-1103P	MT-SO4	MT	MT-5BB	MT-1103P	MT-SO4
**C6 compounds**								
Hexanal	123.97 ± 0.57 A	125.42 ± 6.03 A	ND	ND	262.88 ± 11.28 a	174.84 ± 2.21 c	188.42 ± 2.82 b	181.73 ± 1.22 b
*(E)*-2-hexenal	91.11 ± 0.34 A	79.90 ± 1.90 B	ND	ND	199.41 ± 7.10 a	134.31 ± 2.29 b	142.32 ± 2.14 b	140.79 ± 1.47 b
*(Z)/(E)*-3-hexenol	1.14 ± 0.00 B	1.27 ± 0.51 A	ND	ND	0.90 ± 0.09 d	3.60 ± 0.04 a	1.12 ± 0.04 c	1.38 ± 0.06 b
*(E)*-2-hexenol	14.15 ± 0.72 B	17.85 ± 1.55 A	ND	ND	32.49 ± 0.24 c	41.12 ± 0.63 a	31.42 ± 0.02 d	35.16 ± 0.16 b
Hexanol	16.20 ± 0.02 B	22.27 ± 2.22 A	20.29 ± 0.69 A	21.46 ± 0.06 A	53.47 ± 0.72 c	82.34 ± 1.11 a	62.77 ± 0.19 b	54.77 ± 0.42 c
Hexanoic acid	2.08 ± 0.72 B	2.95 ± 0.13 A	ND	ND	32.43 ± 7.30 b	56.22 ± 1.37 a	39.95 ± 2.37 b	60.72 ± 5.69 a
Isobutyl acetate	ND	ND	2.87 ± 0.12 A	2.81 ± 0.02 A	ND	ND	ND	ND
Butanoic acid, ethyl ester	ND	ND	5.73 ± 0.27 A	5.25 ± 0.00 A	ND	ND	ND	ND
**Monoterpenes**								
Linalool	0.75 ± 0.01	ND	ND	ND	1.58 ± 0.035 c	ND	1.90 ± 0.02 b	2.18 ± 0.03 a
Geraniol	1.81 ± 0.12 A	1.22 ± 0.08 B	ND	ND	6.41 ± 0.20 a	1.79 ± 0.00 b	6.50 ± 0.12 a	ND
Citronellol	0.44 ± 0.00 B	ND	7.29 ± 0.17 A	7.23 ± 0.05 A	ND	ND	ND	ND
**Alcohols**	141.97 ± 0.49 B	154.38 ± 5.21 B	1088.93 ± 42.95 A	1065.72 ± 11.24 A	478.45 ± 16.29 a	458.80 ± 0.90 ab	470.14 ± 3.11 ab	451.02 ± 5.38 b
**Aldehydes**	230.08 ± 0.77 A	217.78 ± 6.90 A	4.67 ± 0.10 B	3.64 ± 0.10 B	494.27 ± 18.21 a	340.68 ± 4.0 1b	357.51 ± 6.13 b	347.98 ± 1.14 b
**Ketones**	10.82 ± 0.04 B	10.52 ± 0.75 B	309.71 ± 9.97 A	4.94 ± 0.04 B	20.83 ± 6.67 ab	11.71 ± 0.15 bc	8.79 ± 0.63 c	22.60 ± 4.79 a
**Esters**	4.93 ± 0.00 B	1.64 ± 0.08 C	11.89 ± 0.47 A	5.89 ± 0.54 B	4.28 ± 0.97 a	1.51 ± 0.21 b	1.45 ± 0.02 b	1.61 ± 0.05 b
**Acids**	6.18 ± 0.12 C	6.27 ± 0.99 C	427.88 ± 16.41 B	684.63 ± 2.86 A	41.96 ± 8.06 b	65.08 ± 2.21 a	45.26 ± 3.87 b	65.66 ± 5.32 a
**Alkanes**	0.68 ± 0.08 A	0.52 ± 0.04 A	0.00 B	0.45 ± 0.01 A	2.62 ± 0.30 a	1.33 ± 0.03 bc	1.14 ± 0.01 c	2.15 ± 0.66 ab
**Alkenes**	0.85 ± 0.22 B	0.83 ± 0.03 B	1.78 ± 0.05 A	1.19 ± 0.09 B	1.95 ± 0.13 b	5.01 ± 1.40 a	1.98 ± 0.22b	1.92 ± 0.29 b
**Total**	395.51 ± 1.63	391.93 ± 17.16	1844.87 ± 85.67	1766.45 ± 17.85	1044.36 ± 41.65	884.12 ± 0.64	886.29 ± 16.6	892.95 ± 9.15

All parameters are listed with their standard deviations (n = 3) (μg·L^−1^). For each parameter, values with different letters are significantly different between the samples (p ≤ 0.05). ND, not detected.

**Table 4:** Volatile compounds of grapes and wines from own-rooted Marselan and Marselan grafted onto different rootstocks: Kober 5BB (MN-5BB) and 1103 Paulsen (MN-1103P) (μg·L^−1^).

	Grape				Wine	
	MN	MN-5BB	MN-1103P	MN	MN-5BB	MN-1103P
**C6 compounds**						
Hexanal	ND	ND	ND	124.28 ± 3.92 b	196.12 ± 0.16 a	204.66 ± 17.47 a
*(E) (Z)*-2-hexenol	ND	ND	ND	31.35 ± 0.97 b	25.65 ± 0.12 c	34.67 ± 1.56 a
Hexanoic acid	ND	ND	ND	26.36 ± 1.12 a	10.59 ± 0.22 c	13.39 ± 0.65 b
*(E)*-2-hexenal	ND	ND	ND	117.51 ± 5.43 b	210.41 ± 1.13 a	201.29 ± 14.27 a
*(E)*-3-hexenol	ND	ND	ND	5.95 ± 0.19 a	4.73 ± 0.00 b	4.66 ± 0.15 b
2-Ethyl-furan	ND	ND	ND	ND	0.66 ± 0.02 a	0.54 ± 0.05 a
Hexanol	22.86 ± 0.14 A	23.46 ± 0.57 A	18.70 ± 0.31 B	52.59 ± 1.44 b	45.40 ± 0.24 c	64.74 ± 2.28 a
Isobutyl acetate	3.34 ± 0.06 B	3.80 ± 0.09 A	2.31 ± 0.01 C	ND	ND	ND
Ethyl ester-butanoic acid	6.44 ± 0.15 A	6.49 ± 0.13 A	6.20 ± 0.11 A	ND	ND	ND
3-Methyl-4-oxo-pentanoic acid	395.60 ± 3.80 B	426.47 ± 10.10 A	372.25 ± 6.89 C	ND	ND	ND
3-Methyl-1-pentanol	0.53 ± 0.00 A	0.37 ± 0.01 C	0.48 ± 0.00 B	ND	ND	ND
**Terpenes**						
Linalool	ND	ND	ND	9.28 ± 0.51 a	9.69 ± 0.15 a	9.62 ± 0.18 a
α-Terpineol	0.53 ± 0.01 C	0.67 ± 0.03 B	1.35 ± 0.02 A	1.05 ± 0.05 a	0.86 ± 0.02 b	0.93 ± 0.00 b
Geraniol	ND	ND	ND	28.92 ± 1.68 b	31.97 ± 0.39 a	26.87 ± 0.27 b
Citronellol	8.14 ± 0.18 C	8.79 ± 0.24 B	9.67 ± 0.18 A	ND	3.96 ± 0.05 a	3.85 ± 0.03 a
Trans-β-ocimene	ND	ND	ND	0.89 ± 0.07 a	0.76 ± 0.18 a	0.72 ± 0.16 a
Neral	ND	ND	ND	0.29 ± 0.01 a	0.25 ± 0.00 b	0.25 ± 0.00 b
β-Myrcene	ND	ND	ND	5.05 ± 0.45 a	5.70 ± 0.42 a	4.95 ± 0.19 a
Farnesene	ND	1.38 ± 0.02 A	0.58 ± 0.01 B	ND	ND	ND
Nerolidol	ND	3.39 ± 0.06 B	5.25 ± 0.11 A	ND	ND	ND
**Alcohols**	1030.39 ± 15.44 A	951.14 ± 23.12 B	839.52 ± 13.05 C	580.09 ± 11.28 a	561.15 ± 5.66 a	546.38 ± 20.56 a
**Aldehydes**	7.93 ± 0.27 A	2.53 ± 0.31 B	0.42 ± 0.34 C	274.43 ± 11.42 b	439.18 ± 0.26 a	433.46 ± 28.19 a
**Ketones**	4.98 ± 0.07 B	5.74 ± 0.13 B	15.18 ± 0.72 A	29.76 ± 4.46 a	27.96 ± 0.53 a	32.62 ± 0.72 a
**Esters**	6.60 ± 0.61 B	10.47 ± 0.29 A	5.13 ± 0.02 C	2.13 ± 0.17 a	0.71 ± 0.51 b	2.05 ± 0.29 a
**Acids**	799.80 ± 30.18 A	798.29 ± 24.26 A	733.09 ± 11.80 B	51.77 ± 17.74 a	18.82 ± 1.51 b	18.28 ± 0.57 b
**Alkanes**	0	0.66 ± 0.54 B	0.96 ± 0.00 A	2.69 ± 0.50 ab	4.05 ± 1.12 a	1.99 ± 0.25 b
**Alkenes**	1.28 ± 0.06 C	2.05 ± 0.06 B	2.95 ± 0.34 A	11.24 ± 1.31 a	12.58 ± 0.02 a	10.98 ± 0.62 a
**Total**	1850.99 ± 56.45	1770.88 ± 57.43	1597.25 ± 32.18	952.10 ± 57.42	1064.46 ± 4.68	1045.77 ± 62.10

All parameters are listed with their standard deviations (n = 3). For each parameter, values with different letters are significantly different between the samples (*p* ≤ 0.05). ND, not detected.

## Data Availability

Data is contained within the article.

## Abbreviations

1103P	1103 Paulsen
5BB	Kober 5BB
AAT	Alcohol acyltransferases
ADH	Alcohol dehydrogenase
DMAPP	Dimethylallyl pyrophosphate
DXR	1-Deoxy-D-xylulose 5-phosphate reductoisomerase
DXS	1-Deoxy-D-xylulose-5-phosphate synthase
FPP	Farnesyldiphosphosphate
G3P	Glyceraldehyde 3-phosphate
GGPP	Geranylgeranylpyrophosphosphate
GPP	Geraniyl pyrophosphate
HDR	1-Hydroxy-2-methyl-2-(E)-butenyl-4-diphosphate reductase
HPL	Hydroperoxides
IPP	Isopentenyl pyrophosphate
LOX	Lipoxygenase
MEP	Methylerythritol phosphate
MVA	Mevalonic acid
SO4	Selection Oppenheim
TDN	1,1,6-Trimethyl-1,2-dihydronaphthalene
TSS	Total soluble solids
